# Improving Dysarthric Speech Segmentation With Emulated and Synthetic Augmentation

**DOI:** 10.1109/JTEHM.2024.3375323

**Published:** 2024-03-11

**Authors:** Saeid Alavi Naeini, Leif Simmatis, Deniz Jafari, Yana Yunusova, Babak Taati

**Affiliations:** KITE, Toronto Rehabilitation Institute, University Health Network (UHN) Toronto ON M5G 2A2 Canada; Institute of Biomedical Engineering, University of Toronto 7938 Toronto ON M5S 3G9 Canada; Department of Speech Language PathologyRehabilitation Sciences Institute, University of Toronto 7938 Toronto ON M5G 1V7 Canada; Hurvitz Brain Sciences ProgramSunnybrook Research Institute (SRI) Toronto ON M4N 3M5 Canada; Department of Computer ScienceUniversity of Toronto 7938 Toronto ON M5S 2E4 Canada

**Keywords:** Dysarthria, speech segmentation, speech recognition, orofacial assessment, data augmentation

## Abstract

Acoustic features extracted from speech can help with the diagnosis of neurological diseases and monitoring of symptoms over time. Temporal segmentation of audio signals into individual words is an important pre-processing step needed prior to extracting acoustic features. Machine learning techniques could be used to automate speech segmentation via automatic speech recognition (ASR) and sequence to sequence alignment. While state-of-the-art ASR models achieve good performance on healthy speech, their performance significantly drops when evaluated on dysarthric speech. Fine-tuning ASR models on impaired speech can improve performance in dysarthric individuals, but it requires representative clinical data, which is difficult to collect and may raise privacy concerns. This study explores the feasibility of using two augmentation methods to increase ASR performance on dysarthric speech: 1) healthy individuals varying their speaking rate and loudness (as is often used in assessments of pathological speech); 2) synthetic speech with variations in speaking rate and accent (to ensure more diverse vocal representations and fairness). Experimental evaluations showed that fine-tuning a pre-trained ASR model with data from these two sources outperformed a model fine-tuned only on real clinical data and matched the performance of a model fine-tuned on the combination of real clinical data and synthetic speech. When evaluated on held-out acoustic data from 24 individuals with various neurological diseases, the best performing model achieved an average word error rate of 5.7% and a mean correct count accuracy of 94.4%. In segmenting the data into individual words, a mean intersection-over-union of 89.2% was obtained against manual parsing (ground truth). It can be concluded that emulated and synthetic augmentations can significantly reduce the need for real clinical data of dysarthric speech when fine-tuning ASR models and, in turn, for speech segmentation.

## I. Introduction

Neurological diseases cause major impairments to oro-motor abilities and can lead to speech impairment (i.e. dysarthria) and/or swallowing impairment (i.e. dysphagia) [1], [2], [3], [4], [5]. Current diagnosis of neurological diseases performed by clinicians often relies on subjective judgement and/or patients’ self-reports, both of which introduce human error and are insensitive to early stages of the disease [6], [7]. This can in turn delay the diagnosis and treatment of neurological diseases at different stages [8], [9].

Limited in-person services and stay-at-home orders during the COVID-19 pandemic motivated clinicians to expedite the utilization of telehealth and remote assessments [10]. This acceleration was especially pertinent given that clinical services and resource allocation related to neurological diseases were already disproportionately scarce, marked by a clear shortage of trained clinicians [11]. In this context, there arises an undeniable need for accessible and automated remote assessments that can objectively detect subtle changes in disease progression, particularly in the early stages of the disease, where timely intervention is more substantive.

A core component of automated speech assessment systems is feature extraction (acoustic from audio and/or kinematic from video). Acoustic features such as speaking rate and pause duration obtained from speech tasks have been shown as valid measures to distinguish neurological diseases at different stages [12], [13], [14]. Kinematic measures of oro-motor control have also emerged as candidate physiological markers of facial bradykinesia in Parkinson’s disease (PD) as well as bulbar signs in amyotrophic lateral sclerosis (ALS). These features are sensitive to early changes, and can provide objective measures regarding particular muscle groups and their corresponding functions [15], [16]. The automatic analysis of kinematic and acoustic features can support objective oromotor structural and functional assessment to track treatment progress in neurological disorders.

A substantial barrier to the adoption of automated speech assessment, particularly in the case of remote assessments, is the considerable post-processing of data samples that is required prior to feature extraction. Given that speech assessments often involve repeating phrases and syllables [17], it is important to segment data into individual repetitions so that features can be extracted [18], [19], [20]. This “parsing” involves counting the repetitions, as well as identifying the onset and offset times of each repetition in the recording (audio or video data). Currently, parsing of audio/video speech data is often performed manually by experienced clinical assistants, making the procedure time-consuming and labour-intensive. Automating the parsing process will contribute greatly to the development of automated and objective oro-facial assessment tools.

Current advancements in the field of machine learning provide opportunities for the development of sophisticated and automated parsing methods. Automatic Speech Recognition (ASR) is a powerful and promising tool in this area. Deep learning architectures, specifically transformer-based models, have achieved state-of-the-art performance in a wide variety of tasks, including ASR [21], [22]. However, the performance of pre-trained ASR models significantly drops in the presence of dysarthric speech [23]. A common practical approach for overcoming this issue is fine-tuning ASR models with representative clinical data. Unfortunately, the fine-tuning process requires large training corpora and the logistical difficulties of clinical data collection and privacy concerns present serious barriers to adoption of these approaches [13], [24].

Factors such as speech variability, articulation, audibility, and accent can manifest different impacts on the performance of ASR for dysarthric speech [25], bringing us to question whether including the data of healthy individuals simulating pathological speech can improve the performance of ASR models. Moreover, research has shown that in facial analysis, synthetic data can be as good, or sometimes even better than real data for training/fine-tuning [26]. Establishing a similar pattern in fine-tuning of audio data would significantly reduce the cost and effort of collecting impaired speech.

In this study, we propose an automatic speech segmentation model that relies on ASR of dysarthric speech. For this purpose, we sought to compare the performance of an ASR system after fine-tuning with various types of relevant speech data. Specifically, we were interested in understanding whether it is a requirement to use pathological voice data to improve the performance of ASR systems on pathological voice samples, or whether it would be possible to attain the same effects using diverse, more-available speech samples either from healthy individuals following standard clinical procedures, or that have been synthesized using text-to-speech (TTS). Particularly, we hypothesize that 1) fine-tuning an ASR system with augmented clinical speech datasets would improve its performance on dysarthric speech, measured using Word Error Rate/WER, and 2) our proposed automatic speech segmentation model will achieve state-of-the-art performance as quantified by intersection over union (IoU) with manually parsed data.

## II. Methods

### A. Participants and Data

We used three different datasets in this study. All three datasets contain repetitions of a sentence “Buy Bobby a Puppy” (BBP), which is a speech task commonly used during an instrumental orofacial examination [27], [28].

The first dataset is a subset of speech recordings in the extended Toronto NeuroFace dataset (TNF) [29]. The original TNF contains video and audio data of 36 individuals performing various orofacial assessment tasks. For the purpose of this study, only audio recordings of the BBP task were analysed. We excluded 5 audio files from the original TNF due to background noise and/or chatter. The dataset was expanded with additional speech data from 37 individuals collected using the same protocol. The extended dataset (TNF ^x^) contained the audio files of 68 participants repeating BBP approximately 10 times (range 9 - 12) at a comfortable speaking rate and loudness. This extended dataset included BBP repetitions from 13 participants with ALS, 27 healthy control participants (HC), 13 post-stroke (PS) participants, 11 participants with PD, 2 participants with primary lateral sclerosis (PLS), and 2 participants with Kennedy’s disease (KD), with a female:male ratio of 29:39. All audio files in this dataset were manually parsed by a trained research assistant to indicate the beginning and end of each BBP repetition. These parsed files were used as the ground truth in this study.

In the compilation of the TNF ^x^ dataset, specific attention was directed towards the delineation of dysarthria subtypes corresponding to the varied neurological conditions represented. For the cohort ALS, dysarthria was predominantly categorized as a mixed spastic/flaccid type, in alignment with typical ALS symptomatology [30]. Patients with PLS were identified as exhibiting primarily spastic dysarthria, consistent with the pathophysiology of PLS [31]. The dataset also includes KD cases, characterized by flaccid dysarthria, reflecting the neuromuscular impairments typical of KD [32]. PD cases within the dataset were characterized as hypokinetic dysarthria as defined in [33] and [34]. The PS subgroup within the dataset presented primarily a flaccid type, but a formal evaluation of the dysarthria types for each of these patients was not performed at this stage. The stratification of dysarthria can be important in evaluating the efficacy of ASR model across a spectrum of dysarthric manifestation, particularly at the more advanced stages of the disease. For the most part, the PS participants presented with a mild dysarthria only.

The second dataset contains the audio recordings of 21 participants aged 18 to 45, with a female:male ratio of 15:6, with no history of speech or other neurological disorders, no cognitive impairments, and representing various ethnicities. Participants in this dataset were asked to repeat the BBP sentence using each the following instructions:

1) at the normal speaking volume and rate,
2) at the normal volume and approximately twice the normal speaking rate,
3) at the normal volume and approximately half the normal speaking rate, and
4) at a loud volume and with the normal speaking rate.

In each case, participants were asked to repeat the BBP phrase approximately 5 times with a short pause between consecutive repetitions (range 4–6 repetitions). This was part of a larger data collection protocol and not all tasks were performed by all participants. We excluded 1 participant from our study due to all BBP files of the person not being available. This dataset was collected online and via a web application (App). More details regarding the complete App dataset can be found in [18].

The third dataset used in this study consists of 3,663 artificially produced human voices using Google text-to-speech (TTS) tool and Tacotron2 [35]. The TTS tool was used to generate synthetic voices of men and women, with 55 various accents repeating the phrase BBP 1–3 times with normal volume and 3 speaking rates: slow, normal, and fast. We only included up to three repetitions to save the cost of training long audio sequences while preserving repetition in speech data.

Table 1 summarizes the number of participants and audio recordings in all three datasets (TNF ^x^, App, and TTS).

**TABLE 1:** Demographic Information of Participants in All Datasets

Dataset	# Participants (Sex F:M)	Total Speech Files	Age range
TNF ^x^	68 (29:39)	68	50 - 93
App	20 (14:6)	80	18-45
TTS	407 (228:179)	3,663	N/A

### B. Automatic Speech Recognition (ASR)

We used audio transcriptions to count the numbers of BBP repetitions in each speech file. To obtain text transcriptions from audio waveforms, we employed the pre-trained as well as fine-tuned Wav2Vec 2.0 (W2V) [21] ASR system. W2V is a framework for self-supervised training that can be broken down into feature encoder, contextualised representation, and quantisation module [36]. The W2V framework exploits the Connectionist Temporal Classification (CTC) [37] loss for training. We used a large W2V model that was trained on pseudo-labeled data [38] and is publicly available in the HuggingFace^1^ repository. We further fine-tuned the model on 7 different subsets of the available datasets to observe whether our augmentation techniques could improve the performance. throughout this study, ‘ADT’ refers to ‘All Disease Types’. The pre-trained model was fine-tuned on

(i) 22 speech files selected from a subset of TNF ^x^ which only included HC data (TNF ^x^–HC),
(ii) 44 speech files from all disease types within TNF ^x^ (TNF ^x^–ADT),
(iii) all speech files from App dataset (App),
(iv) all synthesized speech files from TTS dataset (TTS),
(v) combination of i and iv (TTS + TNF ^x^–HC),
(vi) combination of ii and iv (TTS + TNF ^x^–ADT), and
(vii) combination of iii and iv (TTS + App)

^1^https://huggingface.co

**FIGURE 1.**
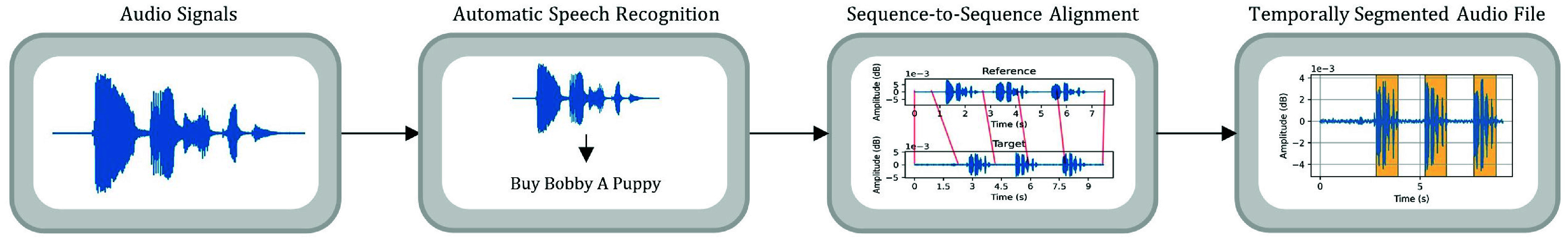
A general overview of our automatic speech segmentation model.

In our study, ASR model’s input consisted of various speech recordings of a single sentence (BBP). These recordings included speech from different individuals, encompassing a range of speaking conditions and neurological states. The primary output of the ASR model was the accurate transcription of these BBP repetitions (as depicted in Figure 1).

### C. Sequence-To-Sequence Alignment

We used Dynamic Time Warping (DTW) [39] to efficiently compute the alignment between two variable-length BBP speech files, i.e. the reference and target. DTW is robust to temporal dilations and shifts of the audio signals and, unlike other approaches like hidden Markov models, does not require careful design and training [40]. The reference speech data was selected from an HC audio file in TNF ^x^ which was excluded from train/test sets. By cropping and concatenating this audio file, we created 13 speech files that had 1 to 13 BBP repetitions. The 13 audio files were then manually parsed. After identifying the correct count using ASR, the corresponding reference file with the same repetition count was aligned with the target speech using DTW.

In order to align reference and target speech, we followed common practice [41] by converting the audio data to their mel frequency cepstral coefficients (MFCC) representation and performed feature matching using DTW. As a pre-processing step, we performed peak normalization on test set audio files, and normalized the MFCC vectors to have a mean of 0, and a standard deviation of 1. The pre-processing step was performed to mitigate the amplitude (loudness) dependency in the reference and target audio, as well as to bring the time-domain signal frames into comparable/similar ranges [42].

In this step, we utilized the transcribed output from the ASR model to quantify the repetitions of BBP. This repetition count was crucial for pinpointing the correct reference audio that needed to be sequentially aligned with the target audio. The result of this sequence-to-sequence alignment process was a set of temporally segmented audio files. Each file was marked with precise start and end timestamps, identifying the duration of each BBP repetition (as illustrated in Figure 1).

### D. Evaluation

The bootstrap resampling is a popular technique in ASR evaluation [43]. This resampling method was used to generate the training and test set pairs. We only selected the test sets from TNF ^x^ as it contains speech files from a variety of neurological diseases and can generalize the performance of the models. We created 3 bootstrap test sets by sampling with replacement. To ensure that train and test sets are balanced, we randomly selected 5 speech files from HC, ALS, PD, PS and included all KD and PLS speech files (2 from each disease category) in each test set; this resulted in train-test split ratio of 65:35.

The performance of each model was evaluated using three metrics: *WER*, to measure the ASR accuracy, *correct count accuracy*, which is the percentage of BBP recordings in which the number of repetitions is counted correctly, and the *IoU*, which measures the alignment between predicted and manually parsed repetitions.

### E. Statistical Analysis

We performed one-way ANOVAs on the values of each of the three metrics (IoU, CCA, and WER) to evaluate whether there were differences between data augmentation conditions. In cases where there were significant main effects, post-hoc testing was performed using Tukey’s honestly significant differences (HSD) test.

## III. Results

Table 2 compares the test performance of pre-trained W2V model versus when it is fine-tuned on various sets containing healthy, pathological, emulated, and synthetic data. The top two best performing models were fine-tuned on 1) TTS + TNF ^x^–ADT and 2) TTS + App.

**TABLE 2:** Evaluation Results of ASRs on Held-Out Test Sets. WER: Word Error Rate. IoU: Intersection Over Union. CCA: Correct Count Accuracy. (Mean ± Standard Deviation)

	WER [%]	IoU [%]	CCA [%]
a) Pre-trained W2V	45.43 ± 2.77	36.94 ± 1.86	43.06 ± 5.20
b) W2V Fine-tuned on TNF ^x^–HC	13.05 ± 1.40	79.59 ± 4.00	72.22 ± 13.75
c) W2V Fine-tuned on TNF ^x^–ADT	9.59 ± 3.73	81.16 ± 0.80	77.78 ± 17.46
d) W2V Fine-tuned on App	6.73 ± 1.10	88.46 ± 1.60	91.67 ± 3.40
e) W2V Fine-tuned on TTS	8.96 ± 5.34	84.28 ± 2.07	86.11 ± 5.20
f) W2V Fine-tuned on TTS + TNF ^x^–HC	7.66 ± 3.05	84.96 ± 2.28	86.11 ± 10.94
g) W2V Fine-tuned on TTS + TNF ^x^–ADT	5.68 ± 0.60	89.15 ± 1.85	94.44 ± 1.96
h) W2V Fine-tuned on TTS + App	5.75 ± 1.02	89.62 ± 1.80	94.44 ± 1.96

Tables 3, 4, 5 show the breakdown of correct count accuracy, WER, and IoU performance per each neurological disease category, respectively.

**TABLE 3:** Breakdown of % Correct Count Accuracy Per Disease Category (Mean ± Standard Deviation)

	HC	ALS	PD	PS	PLS	KD
Pre-trained W2V	66.67 ± 18.86	13.33 ± 9.43	60.0 ± 16.33	46.67 ± 9.43	0.0 ± 0.0	50.0 ± 0.0
W2V Fine-tuned on TNF ^x^–HC	73.33 ± 18.86	73.33 ± 9.43	60.0 ± 32.66	86.67 ± 18.86	50.0 ± 0.0	83.33 ± 23.57
W2V Fine-tuned on TNF ^x^–ADT	93.33 ± 9.43	66.67 ± 24.94	86.67 ± 18.86	73.33 ± 18.86	33.33 ± 23.57	100.0 ± 0.0
W2V Fine-tuned on App	93.33 ± 9.43	100.0 ± 0.0	86.67 ± 9.43	80.0 ± 16.33	100.0 ± 0.0	100.0 ± 0.0
W2V Fine-tuned on TTS	93.33 ± 9.43	100.0 ± 0.0	86.67 ± 9.43	66.67 ± 24.94	66.67 ± 23.57	100.0 ± 0.0
W2V Fine-tuned on TTS + TNF ^x^–HC	93.33 ± 9.43	86.67 ± 9.43	80.0 ± 16.33	80.0 ± 16.33	83.33 ± 23.57	100.0 ± 0.0
W2V Fine-tuned on TTS + TNF ^x^–ADT	93.33 ± 9.43	100.0 ± 0.0	86.67 ± 9.43	93.33 ± 9.43	100.0 ± 0.0	100.0 ± 0.0
W2V Fine-tuned on TTS + App	93.33 ± 9.43	100.0 ± 0.0	86.67 ± 9.43	93.33 ± 9.43	100.0 ± 0.0	100.0 ± 0.0

**TABLE 4:** Breakdown of % WER Per Disease Category (Mean ± Standard Deviation)

	HC	ALS	PD	PS	PLS	KD
Pre-trained W2V	42.40 ± 4.90	48.83 ± 9.27	38.27 ± 7.90	50.0 ± 4.57	67.07 ± 26.27	29.10 ± 11.89
W2V Fine-tuned on TNF ^x^–HC	8.30 ± 1.28	13.87 ± 4.56	14.43 ± 1.32	12.50 ± 7.64	27.80 ± 15.08	7.20 ± 4.17
W2V Fine-tuned on TNF ^x^–ADT	5.97 ± 3.52	9.33 ± 5.05	9.43 ± 6.59	11.73 ± 6.74	20.13 ± 12.43	5.17 ± 5.20
W2V Fine-tuned on App	4.53 ± 2.78	4.30 ± 1.28	8.77 ± 1.37	9.46 ± 6.28	10.57 ± 1.23	3.13 ± 3.18
W2V Fine-tuned on TTS	5.07 ± 1.14	4.13 ± 2.86	8.53 ± 2.19	14.93 ± 10.80	25.27 ± 14.74	0.43 ± 0.61
W2V Fine-tuned on TTS + TNF ^x^–HC	4.90 ± 3.52	3.20 ± 2.15	8.40 ± 1.56	11.87 ± 12.17	20.57 ± 15.58	2.57 ± 2.58
W2V Fine-tuned on TTS + TNF ^x^–ADT	2.13 ± 1.76	2.40 ± 1.10	7.40 ± 4.29	9.10 ± 5.29	15.13 ± 4.81	0.25 ± 0.37
W2V Fine-tuned on TTS + App	3.33 ± 2.24	2.90 ± 1.51	7.93 ± 5.05	8.95 ± 9.38	10.50 ± 0.43	0.37 ± 0.52

**TABLE 5:** Breakdown of % IoU Per Disease Category (Mean ± Standard Deviation)

	HC	ALS	PD	PS	PLS	KD
Pre-trained W2V	39.06 ± 2.13	39.00 ± 3.26	35.00 ± 3.72	32.87 ± 2.57	38.20 ± 1.75	40.81 ± 1.90
W2V Fine-tuned on TNF ^x^–HC	81.24 ± 6.14	83.72 ± 2.08	67.01 ± 5.79	82.99 ± 9.65	82.20 ± 6.70	86.04 ± 5.71
W2V Fine-tuned on TNF ^x^–ADT	90.69 ± 2.32	80.47 ± 0.83	78.69 ± 5.75	76.89 ± 9.52	73.09 ± 1.64	93.81 ± 1.72
W2V Fine-tuned on App	91.93 ± 0.99	92.89 ± 2.04	81.89 ± 5.86	80.85 ± 9.22	90.38 ± 1.73	94.17 ± 2.01
W2V Fine-tuned on TTS	88.96 ± 2.44	91.90 ± 2.18	80.94 ± 6.16	72.28 ± 13.02	88.91 ± 0.97	92.98 ± 2.03
W2V Fine-tuned on TTS + TNF ^x^–HC	89.28 ± 1.71	85.72 ± 3.74	78.19 ± 6.09	80.63 ± 7.50	88.62 ± 1.29	93.20 ± 2.22
W2V Fine-tuned on TTS + TNF ^x^–ADT	92.84 ± 1.89	92.75 ± 3.26	82.91 ± 4.60	85.68 ± 6.78	90.83 ± 1.42	95.03 ± 1.49
W2V Fine-tuned on TTS + App	92.88 ± 1.91	93.77 ± 3.27	83.53 ± 4.76	86.09 ± 6.86	90.88 ± 1.44	95.09 ± 1.52

**FIGURE 2.**
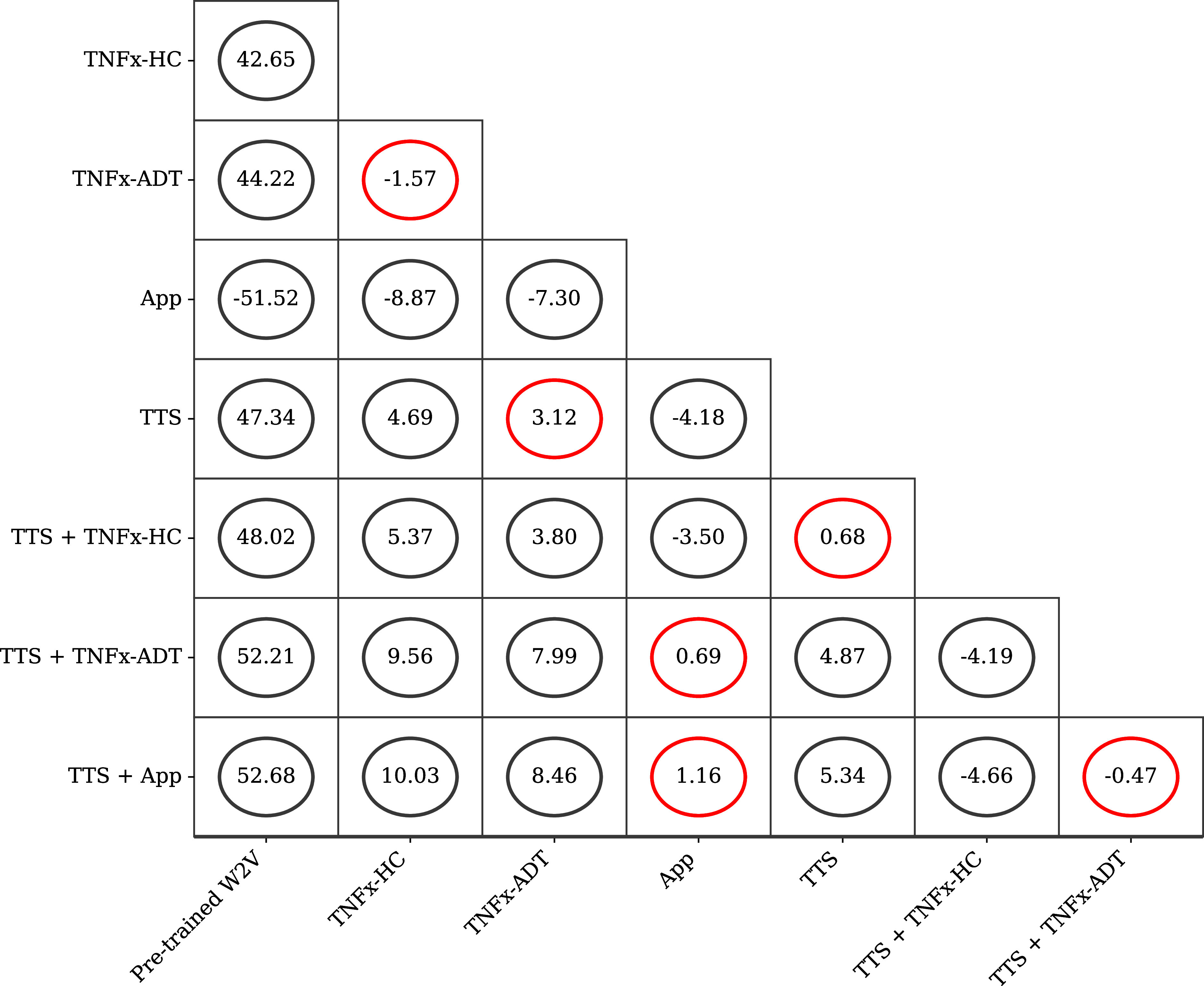
Tukey Honest Significant Differences (HSD) test results for IoU. This figure represents the groups being compared where axes correspond to training sets in table 2. The numbers indicate the mean difference for each comparison, and the circles represent the confidence intervals. The red circles indicate insignificant difference between two groups.

One-way ANOVA test revealed significant differences among the training sets for all metrics, with IoU showing the most substantial variability (( $F-value = 567.80$, $p < 0.0001$), followed by WER ( $F-value = 201.53$, $p < 0.0001$), and CCR ( $F-value=30.75$, $p < 0.0001$). Post-hoc testing using Tukey’s HSD in IoU metric indicated significant pairwise differences between top performing models App ( $mean = 88.46, SD = 1.60$), TTS + TNF ^x^–ADT ( $mean = 89.15, SD = 1.85$), and TTS + App ( $mean = 89.62, SD = 1.80$) and the rest of training sets. For CCA and WER, significant differences were mainly noted between pretrained and fine-tuned ASR models (a and rest of training sets). We concentrated on IoU metric as it represents the performance of our proposed end-to-end model, as opposed to the other metrics that only demonstrate the performance of the ASR portion. Figure 2 shows the mean differences between each pair of training sets with their confidence intervals for IoU metric.

## IV. Discussion

This study demonstrated that combining an ASR model together with a sequence-to-sequence alignment technique, such as DTW, can provide reliable and accurate automatic speech segmentation, leading to strong performance boost. Top performing models in Table 2 (after fine-tuning) achieved IoU values close to 90%. This is a minimum of 25% IoU increase from our previous study [20] which used video data to parse repetitions. These results highlight the value in using representative data to improve ASR performance.

Observing the results in Tables 2–5, using synthetic data alone led to a significant performance improvement, and combining it with a relatively small real dataset led to state-of-the art performance in all disease categories. It is clear that fine-tuning the W2V model using our proposed augmentation methods can significantly improve its ASR performance for dysarthric speech and allow for better extraction of speech features from audio samples with repetition. This work presents an important step toward developing automated remote assessment tools for diagnosis and tracking of neurological disorders, and is significant as there is an urgent need in healthcare to adapt such tools [44].

By taking a closer look at Table 2, the network fine-tuned with control (App) and synthetic (TTS) augmentation data (TTS + App) outperformed the one fine-tuned on only real data, either healthy (TNF ^x^–HC) or real pathological data (TNF ^x^–ADT), and had equal performance when fine-tuned on the combination of real and synthesized speech (TTS + TNF ^x^–ADT). The best models achieved average WER of less than 6%, mean IoU of more than 89%, and mean correct count accuracy of over 94%. This is inline with the statistical analysis performed on training sets where W2V Fine-tuned on App, TTS + TNF ^x^–ADT, and TTS + App showed a statistically significant higher performance compared to the rest of training sets. The results of this analysis indicate the importance of combining dysarthric and augmented data for performance boost.

Our results demonstrate that for the purpose of fine-tuning ASR models and enhancing dysarthric speech segmentation it is beneficial to introduce additional variability into the fine-tuning dataset, in order to improve performance without requiring the complex collection of clinical data for fine-tuning purposes. It is important, however, to note that our approach focused on partial emulation of simple dysarthric symptoms and not on replicating the full complexity of dysarthric speech. This approach aligned with a previous study, where similarly simulated dysarthric speech served as an augmentation strategy [45]. This approach can significantly reduce the need for extensive collections of real pathological speech from individuals with neurological diseases.

Results in Tables 3, 4, 5 show that in 4 out of 5 disease categories, the top two models were able to correctly count the BBP repetitions more than 90% of the time, and that the percentage in the remaining category (PD) was 86.7%. This is inline with previous research [46] as ASR performance in PD is usually poor due to the presence of hypokinetic dysarthria which is associated with quiet, slow, but sometimes rapid, and mumbling speech. In addition, for the top two models, % WER is lowest for KD, HC, and ALS, respectively. The best performing models also had % IoU above 80% in all disease categories, demonstrating high temporal segmentation match with ground truth annotation.

Audio parsing can provide insight into the specific type and severity of dysarthria a patient is experiencing. For instance, the speaking rate, which is a well-accepted objective measure of dysarthria [47], [48], can be estimated by obtaining parsing information. Audio parsing can also be used to segment and analyze recordings of patients performing language tasks that allow for the assessment of various linguistic features, including phonology, syntax, and semantics [49]. These analyses offer insights into the effectiveness of rehabilitative therapies for neurological patients by tracking their progress over time, serving as a valuable tool in the rehabilitation process. As these analyses are automated, they also contribute to the advancements in tele-health and remote assessment of patients.

In this study, we categorized our findings by disease type, recognizing the potential overlaps in dysarthric presentations between different diseases, such as ALS and PS. While we have elaborated on the dysarthria types associated with each disease category in our Methods section, it is important to acknowledge that the TNF ^x^ dataset did not specifically classify speech samples by dysarthria subtype in PS cases. This presents a constraint in our analysis, as it may not fully capture the nuances of subtype-specific dysarthric characteristics. Future work in this field should aim to collect and categorize speech datasets based on detailed dysarthria subtypes to enhance the clinical applicability of ASR models.

A limitation of this study was the analysis of only a single speech task, ‘Buy Bobby a Puppy’, which was utilized as a key element in evaluating our proposed methodology for dysarthric speech analysis. This sentence was selected due to its mix of phonetic components, including both voiced and unvoiced consonants, as well as high and low vowels and diphthongs. It is often used in dysarthria research as it offers some advantages in segmentation (i.e., has easily identifiable boundaries) [18], [50]. While our research focused on this single sentence to enable a clear and controlled comparison of the methodology’s efficacy, we recognize the importance of generalizability in speech analysis. Future research could build upon this foundation by incorporating a broader spectrum of phrases and linguistic variations, thus extending the applicability and robustness of our methodology to a wider range of dysarthric speech patterns.

Another limitation of this study was the use of a single ASR model (i.e W2V). While we acknowledge that comparing our results with newer models could be insightful, we consider this to be a potential area for future research. Extensions of the methods presented in our study could indeed be applied to newer models, providing a valuable direction for subsequent investigations. This current study, however, focuses on demonstrating the significant impact of training data variability and composition on the performance of a well-established ASR model.

Additionally, this study employed a relatively small sample size, which was due to difficulties associated with in-person data collections during the COVID-19 pandemic. Even though the small datasets led to a good performance, there still remains the question of whether there is an optimal amount of data for obtaining best results (and what the optimal amount is). Future studies could answer this by comparing the effect of fine-tuning using different amounts of HC and clinical data to help establish the optimal sample size.

## V. Conclusion and Future Work

This study demonstrated the feasibility of using emulated and synthetic pathological speech as an augmentation strategy to improve dysarthric speech segmentation in people with neurological diseases. Future work will focus on further refining the algorithm, expanding its dataset compatibility, and enhancing its adaptability to a diverse range of speech patterns. These efforts aim to evolve the current framework into a more universally applicable solution, bridging the gap between theoretical development and practical application in dysarthric ASR technology, resulting in minimal real neurological data for training.
